# Supplementary material for: Chromothripsis during telomere crisis is independent of NHEJ, and consistent with a replicative origin
Source: Genome Res. 2019 May;29(5):737–49. doi: 10.1101/gr.240705.118 (PMC6499312; doi:10.1101/gr.240705.118)
Supplement: Supplemental Material [file supp_gr.240705.118_Supplemental_file_1.zip › contigs/annotated_contigs/DB57/contig.2.DB57_length_291_mean_cov_4.37800687285.docx]

**DB57_length_291_mean_cov_4.37800687285**

```
GGTGTGAGCCACCCCACCCAGCCTTAT|CTTATCGTACTTATCGCTCTACCTAAC|ACAGGGTTTCACTCTGTTACCCAGGCTGGAGTG
 >chr17:65271611-65271638 + E=7e-02 p=0e+00            >chr17:65271665-65271867 + E=1e-
CAGCGGTGCAATCATGGTTCACTGCAACCTCTAGCTTCCAAGCTCAAGTGATCCTCCCACCTCAGCCTCCTAAGTAGCTGGGACTGTAG
102
GCATGTGCCACCACACCCAGCTAATTTTTTTTTTTTGAGTGGGAGTCTTGCTCTGTTGCCCAGGCTGGAGTGCAGTGGC|CCCAACTCG

GCGCACTGCATCCTCCGCCGCTCAGGG
```
